# The Influence of the Polymer Type on the Quality of Newly Developed Oral Immediate-Release Tablets Containing Amiodarone Solid Dispersions Obtained by Hot-Melt Extrusion

**DOI:** 10.3390/molecules27196600

**Published:** 2022-10-05

**Authors:** Ancuța Cătălina Fița, Ana Andreea Secăreanu, Adina Magdalena Musuc, Emma Adriana Ozon, Iulian Sarbu, Irina Atkinson, Adriana Rusu, Erand Mati, Valentina Anuta, Anca Lucia Pop

**Affiliations:** 1 Department of Pharmaceutical Technology and Biopharmacy, Faculty of Pharmacy, “Carol Davila” University of Medicine and Pharmacy, 020956 Bucharest, Romania; 2 “Ilie Murgulescu” Institute of Physical Chemistry, Romanian Academy, 202 Spl. Independentei, 060021 Bucharest, Romania; 3 Department of Pharmaceutical Physics and Biophysics, Drug Industry and Pharmaceutical Biotechnologies, Faculty of Pharmacy, “Titu Maiorescu” University, 004051 Bucharest, Romania; 4 Department of Pharmaceutical Technology, Faculty of Pharmacy, “Titu Maiorescu” University, 004051 Bucharest, Romania; 5 Department of Physical and Colloidal Chemistry, Faculty of Pharmacy, “Carol Davila” University of Medicine and Pharmacy, 020956 Bucharest, Romania; 6 Department of Clinical Laboratory and Food Safety, Faculty of Pharmacy, “Carol Davila” University of Medicine and Pharmacy, 020956 Bucharest, Romania

**Keywords:** amiodarone hydrochloride, solid dispersions, hot-melt extrusion, oral immediate release tablets

## Abstract

The present study aims to demonstrate the influence of the polymer-carrier type and proportion on the quality performance of newly developed oral immediate-release tablets containing amiodarone solid dispersions obtained by hot-melt extrusion. Twelve solid dispersions including amiodarone and different polymers (PEG 1500, PEG 4000; PEG 8000, Soluplus^®^, and Kolliphor^®^ 188) were developed and prepared by hot-melt extrusion using a horizontal extruder realized by the authors in their own laboratory. Only eleven of the dispersions presented suitable physical characteristics and they were used as active ingredients in eleven tablet formulations that contain the same amounts of the same excipients, varying only in solid dispersion type. The solid dispersions’ properties were established by optical microscopy with reflected light, volumetric controls and particle size evaluation. In order to prove that the complex powders have appropriate physical characteristics for the direct compression process, they were subjected to different analyses regarding their flowability and compressibility behavior. Additionally, the Fourier transform infrared spectroscopy and X-ray diffraction analysis were performed on the obtained solid dispersions. After confirming the proper physical attributes for all blends, they were processed into the form of tablets by direct compression technology. The manufactured tablets were evaluated for pharmacotechnical (dimensions–diameter and thickness, mass uniformity, hardness and friability) and in vitro biopharmaceutical (disintegration time and drug release) performances. Furthermore, the influence of the polymer matrix on their quality was determined. The high differences in flow and compression performances of the solid dispersions prove the relevant influence of the polymer type and their concentration-dependent plasticizing properties. The increase in flowability and compressibility characteristics of the solid dispersions could be noticed after combining them with direct compression excipients owning superior mechanical qualities. The influence of the polymer type is best detected in the disintegration test, where the obtained values are quite different between the studied formulations. The use of PEG 1500 alone or combined in various proportions with Soluplus^®^ leads to rapid disintegration. In contrast, the mixture of PEG 4000 and Poloxamer 188 in equal proportions determined the increase in disintegration time to 120 s. The use of Poloxamer 188 alone and a 3:1 combination of PEG 4000 and Soluplus^®^ also generates a prolonged disintegration time for the tablets.

## 1. Introduction

Hot-melt extrusion is one of the most widely used processing technologies in the plastics, rubber and food industries. This process is currently applied in pharmaceutical technology for the manufacture of a variety of dosage forms and formulations, such as granules, tablets, suppositories, implants, transdermal systems and ophthalmic inserts [1,2,3].

In addition to being an efficient manufacturing process, hot-melt extrusion improves the quality and effectiveness of manufactured products. The main use of hot-melt extrusion is to disperse the active pharmaceutical ingredients (APIs) into a matrix at the molecular level, thus forming solid solutions [4,5]. In the pharmaceutical industry, it has been used for various applications, such as: (i) increasing the dissolution rate and bioavailability of poorly soluble drugs by forming a solid dispersion or a solid solution; (ii) control or modification of the drug release; (iii) masking the taste of bitter substances; and (iv) formulation of different thin films [6,7,8,9,10].

Hot-melt extrusion has the advantage of being a continuous solvent-free process, that requires few processing steps. It acts like any solid dispersion system by converting the drug from its crystalline form to its amorphous form or by forming a molecular dispersion/solid solution. The amorphous form has more free energy than the crystalline one, thus enhancing the dissolution rate [11,12]. However, the increase in the materials’ free energy affects the thermodynamic stability during storage, leading to the recrystallization phenomenon. Recrystallization will reduce the dissolution rate and solubility of the active pharmaceutical ingredient. Lack of physical stability is one of the main disadvantages of all amorphous dispersions [13,14,15]. For this reason, the need to produce a stable solid dispersion is as essential as the desired improved solubility.

Amiodarone, chemically known as (2-butyl-1-benzofuran-3-yl)-[4-[2-(diethylamino) ethoxy]-3,5-diiodophenyl] methanone, is a benzofuran derivative used to treat supraventricular and ventricular arrhythmias. It is included in the WHO list of essential medicines, being one of the most prescribed antiarrhythmic drugs [16,17].

Its low aqueous solubility (0.2–0.5 mg/mL) and high lipophilic character (class II in the Biopharmaceutical Classification System) lead to problematic absorption [16]. Over time, multiple studies were performed in order to increase amiodarone’s bioavailability, these studies included: self-nano-emulsifying drug delivery systems [18], lipid nanocapsules [19], inclusion in cyclodextrins [20,21,22], nanoparticles [23,24,25,26], and liposomes [27,28]. By including amiodarone in all these new systems, a certain increase in its aqueous solubility was achieved.

Rubim et al. [29] developed amiodarone hydrochloride (AMH)-PEG 1500, 4000 and 6000 solid dispersions. The study proved the solubility enhancement and highlighted the interaction between the drug and PEG 6000 carrier for 1:10 (w/w) formulation. These findings are important steps in establishing the significance of AMH solid dispersions, but they have limitations from a pharmaceutical point of view, as a 1:10 (w/w) ratio leads to an increased amount of active ingredient, and it is impossible to process it under the form of conventional products. Moreover, it is essential to determine the pharmacotechnical properties of the materials, before considering them suitable as ingredients in any pharmaceuticals.

The objective of the present study was to achieve solid dispersions of AMH in a polymer matrix or a mixture of polymers in a precise weight ratio, so that the active substance is solubilized in the polymer melt, and on cooling recrystallizes into an amorphous form with increasing of the solubility. The solid dispersions were then processed into the form of oral immediate-release tablets that must disintegrate in an optimal time to be directly absorbed from the GIT.

In order to obtain AMH solid dispersions in polymer matrices, various 1:2 mass ratio mixtures of the active ingredient with soluble polymers such as PEG 1500, PEG 4000; PEG 8000, Soluplus®, and Kolliphor® 188 were prepared. The influence of the polymer type on the tablets’ performance was established. The physico-chemical characterization of the obtained AMH solid dispersions was performed using Fourier transform infrared spectrometry and X-ray diffraction analyses.

## 2. Materials and Methods

### 2.1. Materials

AMH was purchased from Fagron, Greece, PEG 1500, PEG 4000, PEG 8000, Soluplus^®^, and Kolliphor^®^ 188 were provided by BASF, Germany.

The tablet excipients, the filler-microcrystalline cellulose (VIVAPUR^®^ 302) and the superdisintegrant-sodium starch glycolate (EXPLOTAB^®^), were purchased from JRS PHARMA GmbH & Co. KG, Rosenberg, Germany. The lubricant-magnesium stearate (LIGAMED^®^ MF-2-V) was produced by Peter Graven NV, Netherland.

Commercial tablets of AMH Amiodarone LPH (R) 200 mg, Labormed Pharma SRL Romania were used for comparison.

A Mettler Toledo AT261 balance (with 0.01 mg sensitivity) was used for weighing the ingredients.

### 2.2. Methods

#### 2.2.1. Preparation of AMH Solid Dispersions in Polymer Matrices Using Hot-Melt Extrusion Method

The studied solid dispersions formulations and the temperature used to obtain them are given in Table 1. The quantities are expressed as dose per tablet.

**Figure 1:**
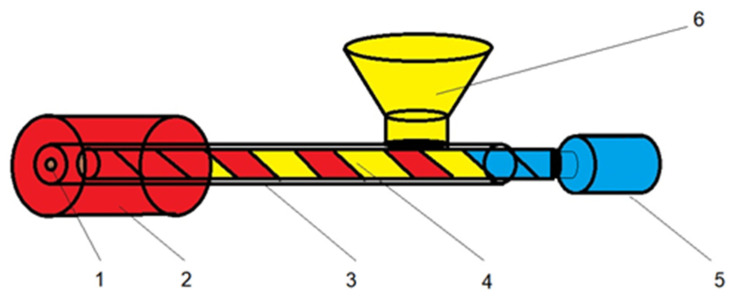
Operating scheme of the horizontal prototype hot-melt extruder (1—extrusion nozzle; 2—heated element; 3—extrusion barrel; 4—extrusion screw; 5—geared motor; 6-loading funnel).

**Figure 2:**
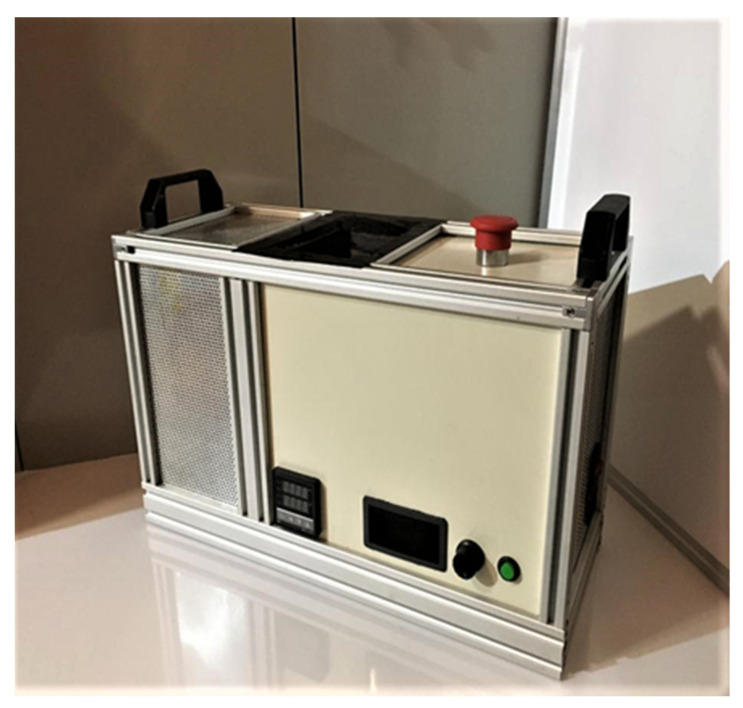
The obtained horizontal hot-melt extruder.

The process was performed using a horizontal prototype hot-melt extruder, developed by the authors in the laboratory. The operating scheme is represented in Figure 1 and the horizontal prototype hot-melt extruder is shown in Figure 2. It consists of a stainless-steel sheath, a 16 mm screw, and a 24 V motor with a variable speed controller. The heating is performed with an aluminum element with high inertia, attached around the stainless-steel barrel, which can generate temperatures from 50 °C up to 300 °C.

The components were weighed according to the mentioned formulations, then fed into the extruder. After 15–20 min, at different temperatures in the 90–140 °C range, the AMH was homogeneously dispersed in the molten matrix of polymers/polymer mixtures. Finally, the dispersion was shaped in filaments by pressing it through the die opening. The filaments were placed onto a waxed support and maintained for 1 h at a 0–8 °C temperature until complete solidification. Immediately, after removing the mixtures from the refrigerator, they were ground in the Quadro Scalable Lab System calibrating impact mill equipped with a round mesh sieve with 1.8 mm diameter and by positioning the cutting parts in the “knife forward” configuration. Care was taken to keep the grinding fast, at a speed of 10,000 rpm, in order to avoid the heating of the rotor and the superficial melting of the granules in the grinding chamber. The obtained pellets were passed through a 1 mm mesh sieve.

Due to the high temperature required to melt for the components of SD12, a brown coloration appeared in the mixture, suggesting a slight degradation of AMH. For this reason, it was decided to stop this experiment, and SD12 was no longer used in the following studies.

#### 2.2.2. Solid Dispersions Characterization

##### Characterization of Solid Dispersions by Optical Microscopy Using Reflected Light

A Motic Panthera microscope with reflected light was used to examine the microstructure of the AMH and solid dispersions. The use of tangential illumination was chosen in order to easier observe the samples. The observation of the melting phenomenon or melt solubilization of the active substance in the polymers/polymers matrix was sought.

##### The Assessment of Solid Dispersions Volumetric Characteristics

In order to assess the behavior of a material in the compression process, it is essential to study its performance in a mechanical compaction procedure. The initial volume and the volume after compaction give indications of the powders’ property to diminish their volume under the action of a mechanical force, and the particles’ ability to occupy the free spaces in its bulk structure. The more similar the volumes are, the substrate was better arranged in the constrained space of the cylinder, resulting in the conclusion that the powder has good flowability, but it may indicate a lack of compressibility [30,31].

One of the parameters that gives an indication of the solid material’s flowability and compressibility is the Hausner ratio (HR), obtained from the ratio between the density at compaction and the apparent density of the powder. A high value of this index indicates an accentuated cohesive character of the powder and a diminution of the free flow.

Another parameter for measuring the compressibility is the Carr index (CI), which is also calculated from the values of the initial and tapped densities, according to Equation (1).

(1) $$CI\left(\%\right)=100\times \frac{\left(\rho_{tapped}-\rho_{bulk}\right)}{\rho_{tapped}}$$

where *ρ*_*tapped*_ is the tapped density of the material (kg/m^3^) and *ρ*_*bulk*_ is the initial bulk density of the material (kg/m^3^).

Bulk density or volumetric mass is an important characteristic for powder rheology, along with the bulk volume, pore volume and porosity [32].

According to European Pharmacopoeia (EPh) [33], the relationship between percentage compressibility (CI) and powders flowability is indicated in Table 2.

The study was performed using the Vankel Tap Density Tester, produced by Vankel Industries Inc., USA. Bulk and tapped density, HR, and CI were calculated. Then, 20 g of each sample were poured into the graduated cylinder and the bulk volume was read. Successive 10, 500 and 1250 mechanical shocks are caused and the corresponding V_10_, V_500_, V_1250_ volumes are measured. If the difference between V_500_ and V_1250_ is more than 2 mL, the sample is again subjected to another 1250 shocks, and the resulting volume is read (V_2500_). Five determinations are made for each powder and the average is calculated [34].

##### Particle Size Evaluation

Powders with a small particle size have a larger contact surface, high compressibility, and a higher dissolution rate, but poor flowability. In practice, a compromise between these aspects needs to be made [35].

The granulometric analysis of the samples and the cumulative distribution were carried out by the method of sieving by mechanical shaking, with CISA Sieve Shaker Mod. RP 10, produced by CisaCedaceria Industrial, Spain. Next, 20 g of each sample were transferred to the first sieve, then the system was shaken for 15 min to a 2.5 mm amplitude. At the end of the scheduled time, the remaining fractions on each sieve and in the collection container at the base of the assembly are weighed.

##### FTIR Analysis

Fourier Transform Infrared spectroscopy was performed on a NICOLET 6700 FT-IR spectrophotometer (Thermo Electron Corporation) in the range of 400–4000 cm^−1^, in transmittance mode.

##### XRD Analysis

Powder X-ray measurements were recorded using a Rigaku Ultima IV diffractometer (Rigaku Co., Tokyo, Japan) in parallel beam geometry using a CuKα radiation (wavelength 1.5406 Å). The XRD patterns were recorded in 2θ range between 5° and 60° with a speed of 2°/min and a step size of 0.02°.

#### 2.2.3. Complex Powders for Direct Compression Containing AMH Solid Dispersions

As already mentioned, the final aim of the study was to manufacture oral immediate-release tablets containing the AMH solid dispersions. For obtaining the tablets, the direct compression method was selected, as it is considered the most advantageous technology due to the lack of humidity, the high stability, and the fastness of the process. In order to achieve high-quality tablets, the direct compression blend must present suitable properties, making the choice of the excipients and the formulations of complex powders the critical steps in the development process [36,37].

The prepared solid amiodarone dispersions will be the active ingredients of oral tablets. The physicochemical properties of the particles (such as shape, size, density, weight) as well as the mechanical properties (elasticity, plasticity, fragility) influence the behavior of the blends during the compression process and subsequently as drugs. 

##### Powders Formulation and Preparation

The compression excipients have been selected to ensure good compactness and superior disintegration. Thus, the mixtures were diluted with microcrystalline cellulose and homogenized with the sodium starch glycolate superdisintegrant. As solid dispersions are known to have a tendency to stick to the surfaces of the punches, it was decided to use a magnesium stearate with a large contact surface (Ligamed MF-2V, Peter Greven). The excipients amounts were selected in order to obtain tablets containing 50 mg amiodarone hydrochloride/tablet [38,39].

Eleven formulations were performed, according to the corresponding obtained solid dispersions. All powders for direct compression are containing 30% AMH SD, 68% microcrystalline cellulose, 1% sodium starch glycolate, and 1% magnesium stearate.

The obtained solid dispersions obtained were homogenized at room temperature, for 10 min, with the excipients for direct compression, followed by lubrication of the mixtures with magnesium stearate for another 5 min, in a rotating device at a speed of 14 rpm.

##### Evaluation of the Powders’ Physical Characteristics

One of the essential objectives for the manufacturing of the tablets is to ensure the free flow of the material, which in turn determines: (i) the uniform loading of machine molds, (ii) the uniformity of mass and of the physical–mechanical properties of the tablets, (iii) avoiding the air incorporation into the powder bed and thereby avoiding undesirable phenomena that may occur in the preparation of tablets, such as pickling or lamination, and (iv) limiting the formation of fine particles in the powder mass, which could lead to increased friction with the walls of the mold, preventing proper lubrication [40,41,42].

The final powders were analyzed, and their degree of fineness, flow and volumetric properties were determined using the methods already mentioned.

#### 2.2.4. Oral Immediate-Release Tablets Formulation, Manufacturing and Quality Assessment

##### Tablets Formulation

Following the data obtained in the preformulation step, after determining their physical properties, it was found that all solid dispersions can be processed into tablets by direct compression.

The final tablets formulations are shown in Table 3.

##### Tablets Manufacturing

The complex powders were compressed with a 30 N force, in a Korsch EK-O type single-post eccentric machine, equipped with 12 mm flat punches. 

All series of tablets were tested according to European Pharmacopoeia specifications [33].

##### Tablets Quality Characteristics

*Pharmacotechnical properties*—dimensions (diameter and thickness), mass uniformity, hardness and friability

The tablets’ pharmacotechnical attributes provide useful information on the type of deformation that occurred in the powder bed at the time of compression and indicate whether the transformations were of a plastic or elastic nature. Moreover, suitable mechanical characteristics ensure the biopharmaceutical performance of the tablets [43].

The diameter, thickness, mass uniformity and hardness were determined using an Erweka TBH-30 combined tester, produced by Erweka® GmbH, Germany. Ten tablets of each formulation were analyzed for each characteristic.

The friability was also performed on 10 tablets of each formulation, using the Vankel friabilator, at a rotating speed of 25 rpm for 5 min. The loss in weight is calculated and expressed as a percentage. The acceptable values are not more than 1.0 %. 

##### Biopharmaceutical Properties In Vitro Disintegration Time and Dissolution Rate

In vitro disintegration time was assessed according to EPh 10 [33], on 6 tablets of each formulation, in distilled water at 37 ± 0.5 °C, with Erweka DT 3 apparatus, produced by Erweka® GmbH, Langen, Germany.

In vitro dissolution rate was evaluated using USP paddle Apparatus II (dissolution tester Hanson Vision G2 Classic 6, produced by Teledyne Hanson Research^®^, Langen, USA), in 1000 mL 1% (*w*/*v*) sodium dodecyl sulphate, at 37 ± 0.5 °C, with a paddle rotating speed of 100 rpm. The AMH dissolved amount was determined at 15, 20, 30, 45, 60 and 90 min, by a in house developed UV-HPLC (LC-4000 Series HPLC system (Jasco) at the absorption maximum of 242 nm [44]. Column: Kinetex^®^ 2.6 µm C18 100 Å, 100mm × 3 mm, Phenomenex, Column temperature: 30 °C; Mobile phase: Solvent A: 0.1% formic acid, Solvent B: Acetonitrile: Methanol (1:1 *v*/*v*); Mobile phase composition: Solvent A/Solvent B = 20:80; Flow rate: 0.7 mL/min. Detection: UV; analytical wavelengths: λ = 242 nm; Injection volume: 5 µL. The dissolution profiles for each formulation were registered.

Dissolution profile comparison was performed based on the f2 similarity factor [45]. Since f2 value is sensitive to the number of dissolution points taken into account for calculation [46], the similarity factor was calculated up to 45 min, the first time point where the dissolution exceeded 85% amiodarone released for all evaluated formulations.

## 3. Results

### 3.1. Solid Dispersions Characterization

SD 1-11 lead to smooth, opaque-white extrudates, but SD 12 filaments had a brown coloration, the reason for which it was removed from further studies. All solid dispersions obtained were easily ground. White, uniform powders in appearance, with high density, resulted. Compared to the active substance which has a gritty nature, the SDs obtained have a glossy appearance, with optimal granulation.

#### 3.1.1. Characterization of Solid Dispersions by Optical Microscopy Using Reflected Light

**Figure 3:**
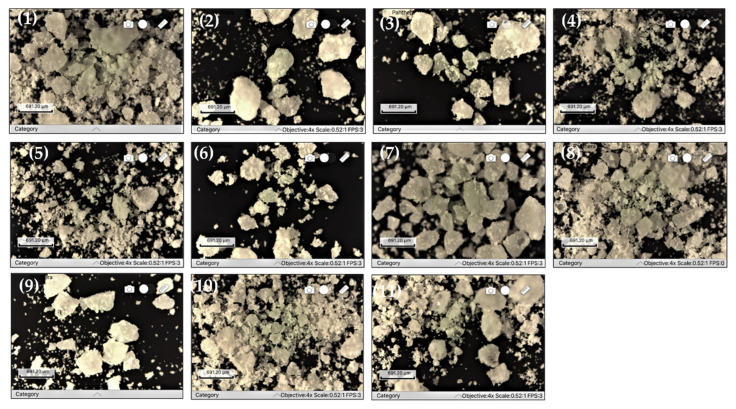
SDs micrographs (**1**) SD1; (**2**) SD2; (**3**) SD3; (**4**) SD4; (**5**) SD5; (**6**) SD6; (**7**) SD7; (**8**) SD8; (**9**) SD9; (**10**) SD10; (**11**) SD11.

**Figure 4:**
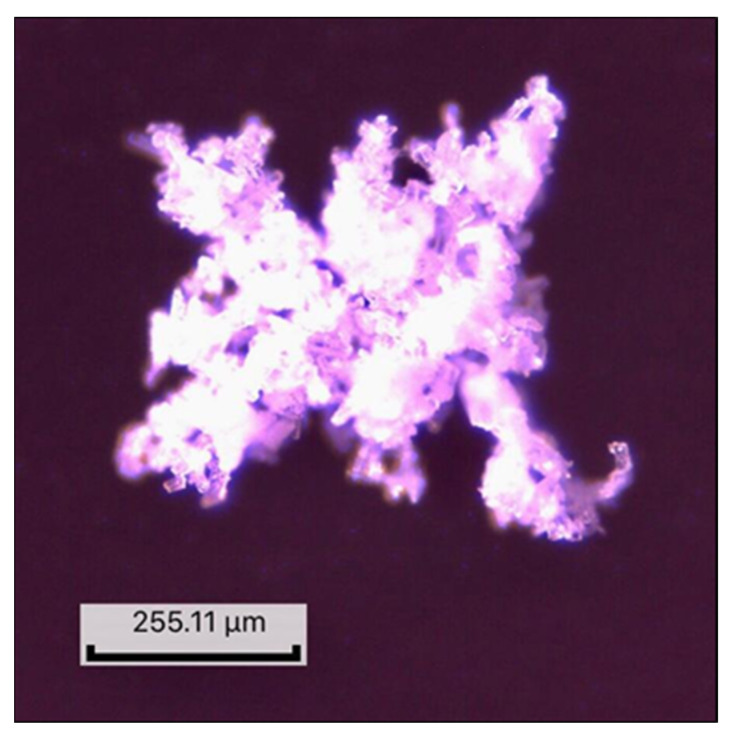
AMH micrograph.

The recorded microscopic images for all 11 SD are given in Figure 3 and for active substance the micrograph is given in Figure 4.

The optical micrographs revealed a similar morphology for all obtained compounds, with heterogeneous shapes and multimodal size distribution. Although the surfaces are irregular, the particles display a translucent appearance, leading to the conclusion that AMH is homogeneously dispersed in polymer matrices. The wide variety of the particles’ dimensions will influence their flowability and compressibility, these analyses being mandatory under these conditions.

#### 3.1.2. The assessment of Solid Dispersions Volumetric Characteristics

The results regarding the volumetric characteristics of the solid dispersions are given in Table 4.

In all cases of the studied powders, the decrease in the volumes is more accentuated during the first 500 mechanical shocks, then the changes are insignificant. Concerning the volumetric characteristics, the compounds exhibit significant differences between formulations. The only batch presenting an excellent free flow is SD4 which contains equal quantities of AMH, PEG 8000 and Poloxamer 188. SD1, SD 5, SD6, SD7, SD9 and SD11 show good flowability, enough for their subsequent processing by direct tableting. Moreover, SD2, SD3, SD8 and SD10 are displaying only a fair flow behavior, making more important the influence of the tablets’ excipients in the compression process. The results are surprising, knowing that the hot-melt extrusion solid dispersions are usually sticky and not characterized by a free flow [47,48]. Still, none of the studied formulations had a poor flow, proving the accurate selection of the polymers, as well as the used amounts.

The solid dispersion formulations with PEG as the majority ingredient show a better flow than those combined with Soluplus^®^, probably indicating a higher cohesion between the particles in SD7–SD11.

#### 3.1.3. Particle Size Evaluation

**Figure 5:**
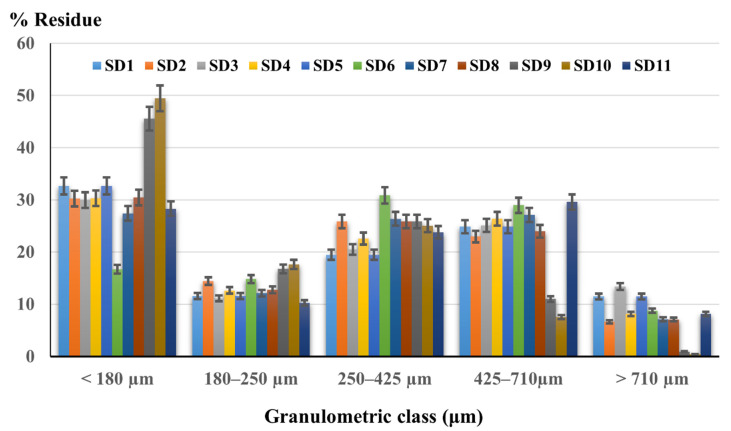
Granulometric analysis of solid dispersions.

Figure 5 shows the histogram registered by representing the particle size distribution on granulometric classes for all eleven studied solid dispersions.

There are no significant differences in the particle size distribution between the eleven types of solid dispersions, the lowest percentage being registered for the particles larger than 710 µm. SD9 and SD10 have almost half of the particles less than 180 µm but they did not seem to have an important influence on the flowability of the powders. Regarding the below 180 µm class, fractions of 28–32% fine particles were presented in the other formulations. For 250–425 µm and 425–710 ranges µm, most of the dispersions recorded percentages between 20 and 30%.

#### 3.1.4. FTIR Analysis

**Figure 6:**
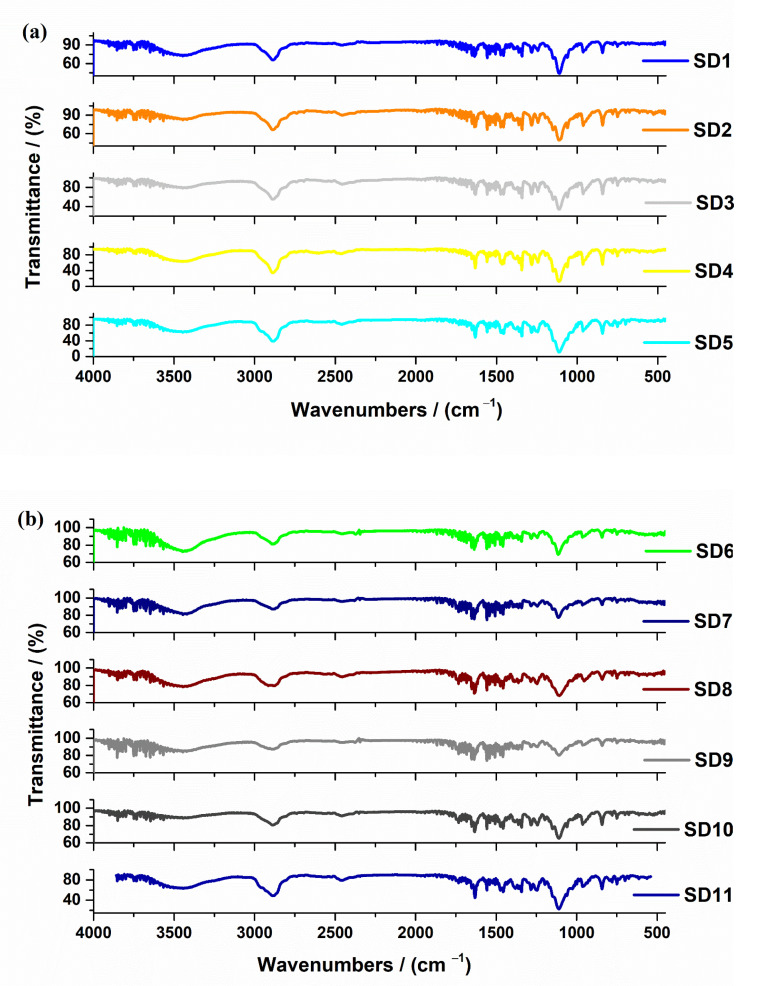
FTIR spectra of studied solid dispersions (**a**) SD1–SD5; (**b**) SD6–SD11.

The FTIR spectra of all eleven solid dispersions are represented in Figure 6a,b.

According to data in the literature, Soluplus® showed peaks at 3448 cm^−1^ due to O–H stretching, at 2927 cm^−1^ due to aromatic C–H stretching, at 1735 cm^−1^ and 1635 cm^−1^ due to C=O stretching, and at 1477 cm^−1^ due to C–O–C stretching. All these peaks are observed in the FTIR spectra of studied solid dispersions which contain Soluplus^®^ (SD7–SD11) [49,50].

The FTIR spectra of AMH show the presence of C–H stretching vibration bands from methyl groups at 2977, 2963 and 2858 cm^−1^, the C–H stretching from methylene groups at 2933, 2918 and 2880 cm^−1^. At 1477 cm^−1^ (asym) and 1381 cm^−1^ (sym) appear C–H deformation vibrations from C–CH3 bonds, while for those from the methylene group, they appear at 1454 and 1432 cm^−1^. At 3040 cm^−1^, the =C–H appears to stretch from the aromatic structure, while in 900–860 spectral range the in-plane deformation of the 1,2,3,5-tetrasubstituted ring appears. The band which appears at 1629 cm^−1^ is due to the characteristic ketone band from diaryl ketone, while the aryl–alkyl ether band and the one from benzofuran ring appear at 1285 and 1246 cm^−1^, respectively. The bands which appear at 1790 and 1560 cm^−1^ are due to the NH^+^ vibrations. These bands are in good agreement with the chemical structure of the AMH compound [51]. 

According to data in the literature, the FTIR spectrum of poloxamer 188 is characterized by principal absorption peaks at 2883 cm^−1^ attributed to C–H stretch aliphatic, 1341 cm^−1^ attributed to in-plane O–H bend, and 1102 cm^−1^ attributed to C–O stretch. All these bands are shown in Figure 2 for SD3, SD4, SD5 and SD11 solid dispersions [52]. 

PEG shows important characteristic absorption peaks at 3426 cm^−1^ and 1102 cm^−1^ which are attributed to the O–H stretching vibration and the symmetrical stretching vibration of C–O–C, respectively, and the characteristic absorption peaks of the C–H stretching bonds at 2889 cm^−1^, 1460 cm^−1^, 951 cm^−1^, and 843 cm^−1^. A slight difference between the peaks’ values is due to the use of the different type of PEG: PEG 8000, PEG 4000, or PEG 1500 [53].

FTIR spectroscopy analysis was used to verify the type of interactions between AMH and the used excipients in all solid dispersions. According to the literature, the type of bonding between the used excipients and AMH was hydrogen bonding [54]. The reduction in peak intensities for the aromatic C–C ring and the ketonic C=O binding of AMH in the FTIR solid dispersions spectra, even more the peaks shift or disappearance of the bands between 2700–2200 cm^−1^, indicates the possible interactions between the compounds from the solid dispersions.

#### 3.1.5. XRD Analysis

**Figure 7:**
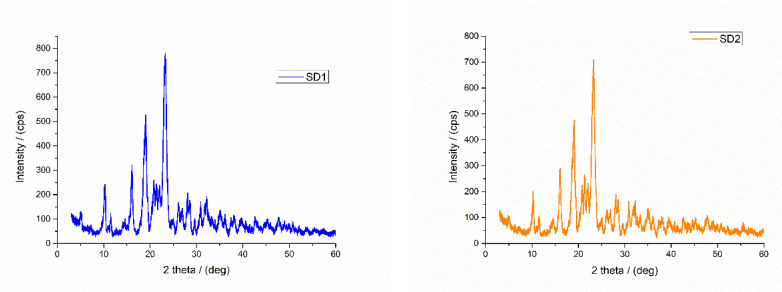

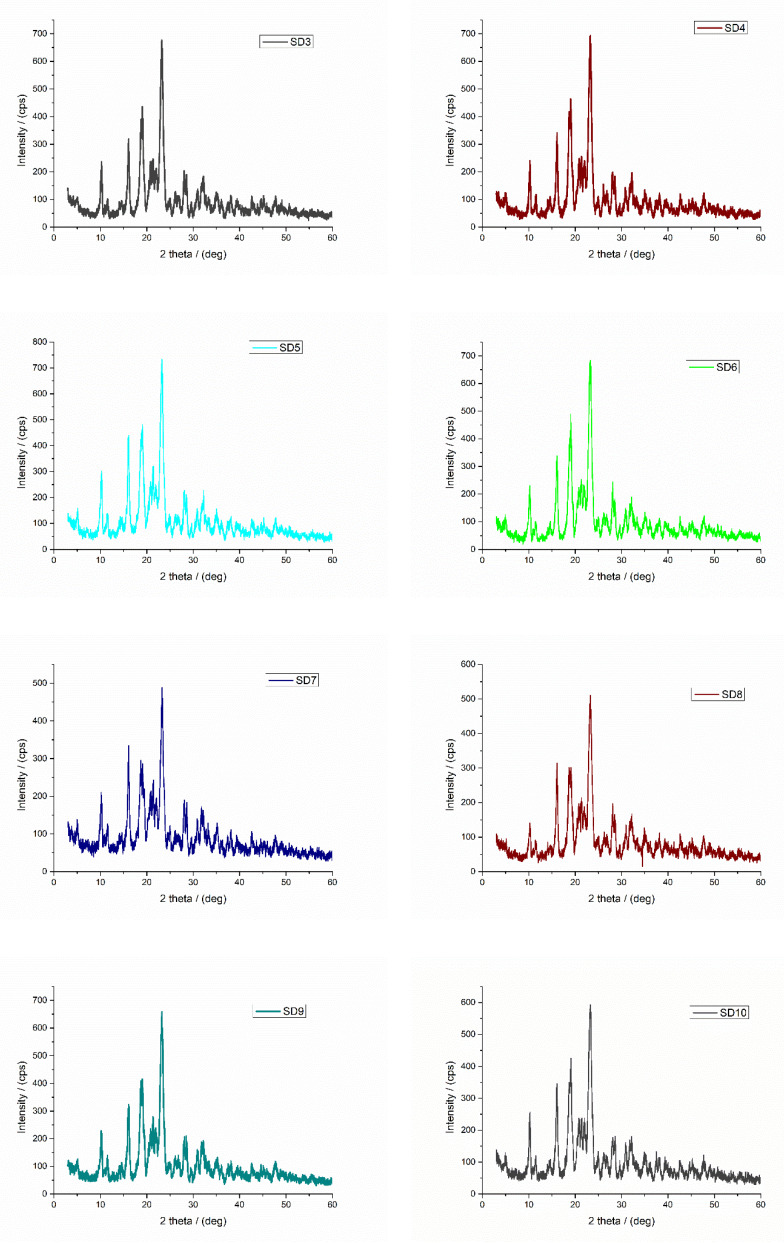

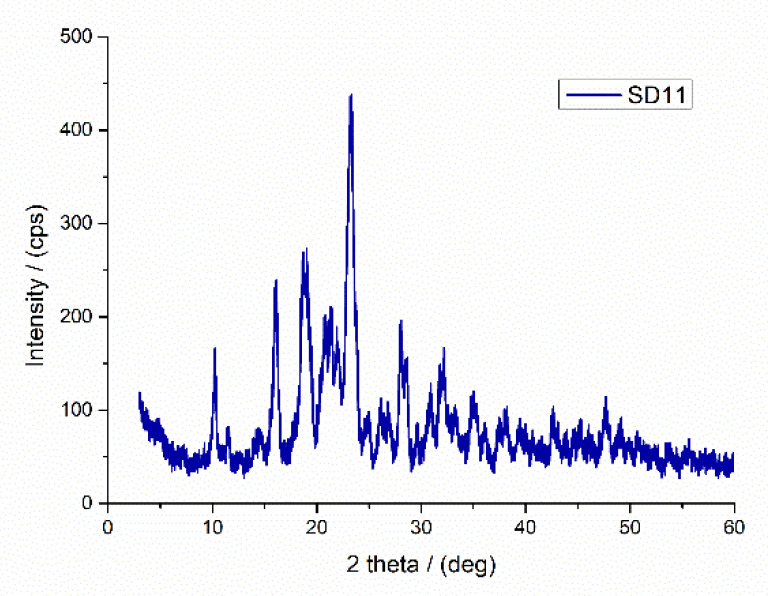
X-ray diffraction patterns of solid dispersions.

The XRD patterns of solid dispersions are shown in Figure 7. From the literature, the diffractogram of Soluplus® displayed no peaks due to its amorphous nature [50]. The poloxamer 188 presented two peaks at 19.3 and the other broader one between 22° and 23° [55].

PEG have two strong and sharp diffraction peaks at about 19.6° and 23.4° [56,57]. The XRD diffractograms from Figure 7 show the crystalline nature of the studied solid dispersions. According to literature, the XRD pattern of pure AMH is characterized by intense crystallinity peaks which are observed at 2θ = 5.2°, 7.3°, 10.3°, 11.6°, and 16.1°, corresponding to (100), (110), (020), (210), and (211) planes (ICDD no. 00-045-1755) [58]. In all solid dispersion, the intensity of XRD peaks characteristic of the drug decreased and some XRD peaks were suppressed with those of the used excipients, thus indicating a reduction in drug crystallinity.

### 3.2. Complex Powders for Direct Compression Containing AMH Solid Dispersions

After mixing the solid dispersions with the selected excipients for compression, eleven white and uniform powders were obtained. The powders have resembling appearances and were all studied in order to establish their physical properties.

#### 3.2.1. Evaluation of the Powders’ Physical Characteristics

##### Volumetric Characteristics

In Table 5 are shown the values obtained for specific volumetric parameters.

The increase in flowability and compressibility characteristics of the solid dispersions can be noticed after combining them with direct compression excipients owning superior mechanical qualities. F4 and F11 are remarkable for their excellent free flow, while all the other formulations exhibit good physical attributes.

##### Particle Size Distribution

**Figure 8:**
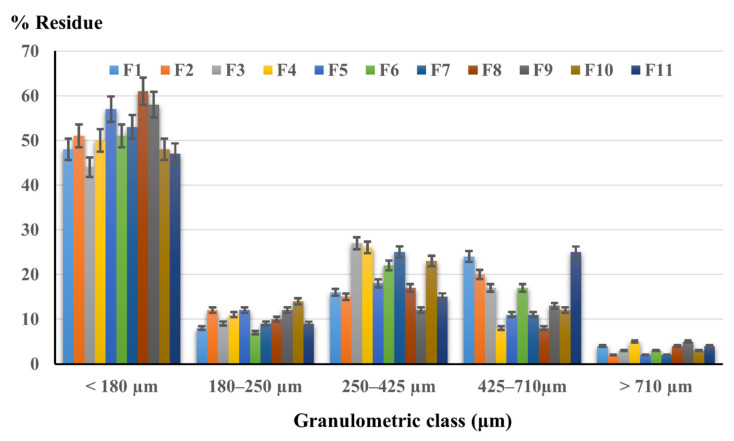
Particle size distribution for direct compression powders.

The registered granulometric histogram is shown in Figure 8.

Concerning the particle dimension, a drastic change can also be observed after mixing the solid dispersions with direct compression excipients. Similar to the active ingredients’ outcome, the particles of complex powders display a uniform size distribution between all formulations. Still, in compression blends, most of the particles lay below 180 µm and the large particles are in lower proportions.

### 3.3. Oral Immediate-Release Tablets Quality Assessment

All formulations lead to the manufacturing of white, round-shape, compact, homogeneous, with smooth, uniform surfaces, and intact edges tablets.

The pharmacotechnical properties of all tablets’ batches are given in Table 6.

According to the recorded results, the uniformity of the tablets sizes and weights between all formulations is obvious. The tablets’ diameter varies from 11.9 mm to 12.1 mm, the thickness is in the 4.02–4.16 mm range and the average weight fluctuates between 492.1 mg and 506.3 mg, thus all tablets’ series are meeting the requirements of the EPh [33]. F8, which contains PEG 1500 and Soluplus^®^ in a 3:1 mass ratio, presents the lowest sizes and weight. Somehow, this could have been predicted considering that it was the formulation with the lowest tapped density (0.41 g/mL). On the other hand, the tablets manufactured according to F11, containing a 3:1 mass ratio of Poloxamer 188–Soluplus^®^ polymer mixture, have the highest sizes and mass, and F11 also exhibited excellent flowability in the previous tests. This indicates the influence of the polymer/plasticizer type on the direct compression blends behavior during the tableting process.

Regarding the hardness of the tablets, more variability in the results is noticed. It varies from 78 N (F8) to 96 N (F2), even the applied compression force was the same. The friability was low, and corroborating the results of both mechanical resistance tests, it is clear that all series of tablets will maintain their integrity during future handling. Despite the differences between the formulations, all hardness values are according to the standards imposed in pharmaceutical practice.

Concerning the tablets’ disintegration time, significant differences are observed between formulations. The results fall into a wide range of 10 to 160 s. Indeed, all tablets’ batches present a suitable disintegration time that is in accordance with the regulations imposed by EPh [33], but the variations of these values will highly impact in vivo performance of the tablets. It is interesting that a correlation between the tablets’ hardness and their disintegration time cannot be found, as the exact three formulations (F3, F5 and F9) that possess the lowest resistance have the highest disintegration times, of 120 and 160 s. F6 and F8 disintegrate in 10 s, which is an excellent and ideal time for any oral immediate-release tablets.

The release profiles look quite similar for all formulations and the dissolution rate after 60 min is in accordance with pharmacopeial requirements [44] for all eleven series, ranging between 91.7% (F9 based on PEG 4000 and Soluplus^®^ in 3:1 mass ratio) and 97.66% (F4 which contains PEG 8000 and Poloxamer 188, in equal quantities). This was observed for the commercial product also.

F4 release performance could have been predictable as it also presented a low disintegration time, of 40 s. Still, after 15 min, F4 released only 51.05% of its AMH content, in comparison to 76.23% for F6. F6 displayed the lowest disintegration time (10 s), but after 60 min it released 95.12% of AMH, less than F4. The lowest dissolution rate after the first 15 min belongs to F7 (41.72%) containing PEG 1500 and Soluplus^®^ in a 1:1 mass ratio, but it reached 93.44% after 60 min. This was unexpected considering its fast disintegration (25 s). However, the remarked differences are due to the amount or type of polymer used to obtain the solid dispersions.

When focusing on the first 45 min release (in accordance with *f*2 similarity requirements), the data show differences between the formulations themselves, and between SD formulations and the commercial product.

**Figure 9:**
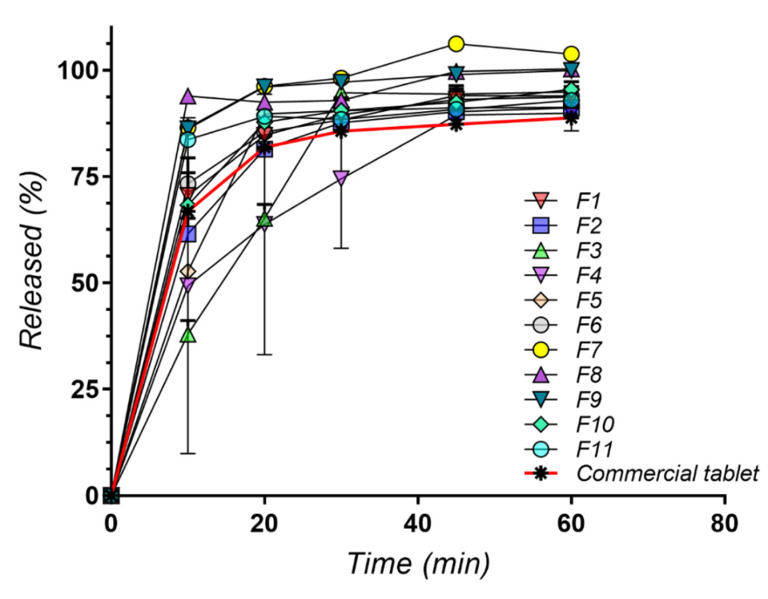
Dissolution profiles for all series of tablets.

The dissolution profiles of AMH SD tablets and the commercial product are presented in Figure 9.

**Figure 10:**
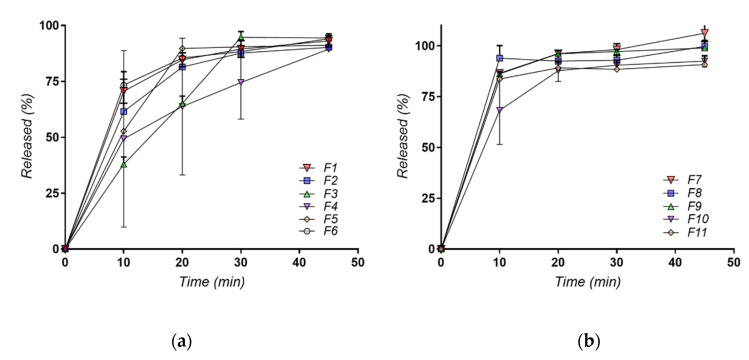
Dissolution profiles for formulations (**a**) F1–F6 and (**b**) F7–F11.

Comparison between formulation F1–F6 (mixtures of amiodarone with PEG and poloxamer) and F7–F11 formulations (mixtures of Soluplus with polymers) showed an increase in the initial dissolution rate in the first 10 min for the formulations with Soluplus^®^ (Figure 10a,b). This is a good indicator of formulation performance taking in consideration that in oral immediate-release tablets formulation the first 15 min plays an important role in the bioavailability of the product.

**Figure 11:**
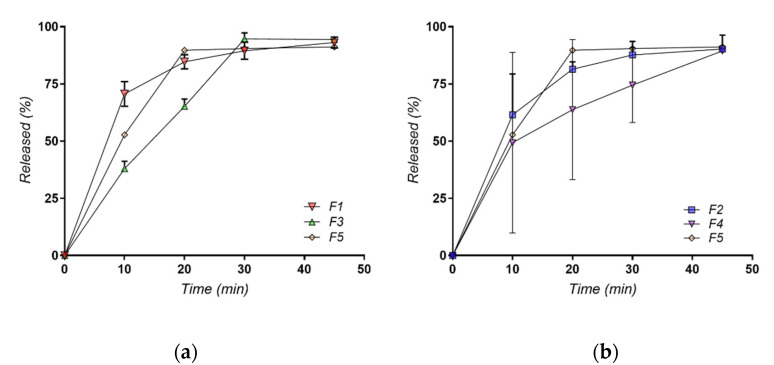
Dissolution profiles for formulations (**a**) F1, F3, F5 and (**b**) F2, F4, F5.

Formulations with AMH mixtures with polymers showed that comparing the dissolution rate between the solid dispersion of AMH in simple matrix of PEG4000 or Poloxamer 188 showed a faster profile compared with the mixture of AMH in a mixture of 50/50 PEG4000 or Poloxamer 188. The same observation was showed comparing PEG 8000 and Poloxamer 188 alone compared with AMH in a mixture of 50/50 PEG4000 or Poloxamer 188. This can be observed in Figure 11a,b.

Similarity *f*2 factor was calculated for all the formulations, and a matrix of similarities was made to better observe the similarities and differences between all the formulations. 

It can be observed from Table 7 that formulations F11–F10 are the most similar with the rest of the formulations and they also have a good similarity factor compared with the commercial product.

From the groups of Poloxamer and PEG (F1–F6) and Soluplus® group (F7–F11) a special interest presented F6 and F9. They showed in their groups the highest and fastest profile release of AMH. Based on *f*2 factor formulation F6 and F9 are border line similar with an *f*2 of 50.45, although when we compare them with the commercial product if F6 is similar with the commercial product at a value of *f*2 at 63.78 (grater then 50) on the opposite F9 shows a dissimilarity with a *f*2 value of only 41.81.

**Figure 12:**
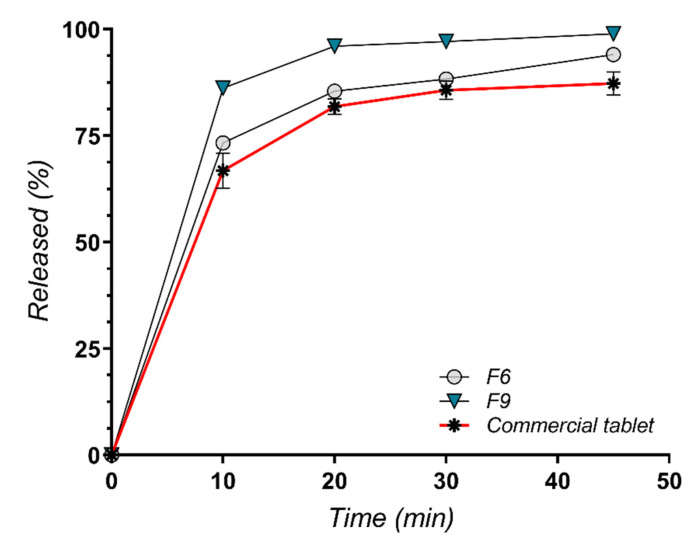
Dissolution profiles for formulations F6, F9 and commercial preparation.

Figure 12 shows that the best improvement in dissolution rate and profile is for the formulation F9 that uses a mixture of Soluplus and PEG4000 at a ratio of 25:75. Additionally, the RSD of the formulation F9 was better than the commercial formulations.

## 4. Discussion

For extrudates manufacturing, PEG 1500, PEG 4000, PEG 8000, Soluplus^®^, and Kolliphor^®^ 188 were selected as soluble matrix-forming polymers. The selection of the polymers is a crucial procedure in the development of solid dispersions. For SD 7–12, Soluplus^®^, which is an amphipathic graft copolymer consisting of polyvinyl caprolactam, polyvinyl acetate and polyethylene glycol (13% PEG 6000/57% vinyl caprolactam/30% vinyl acetate) was selected. It has the benefit of a low glass transition temperature (around 70 °C) and good thermal stability (up to over 290 °C) [59]. Soluplus^®^ proved to be a suitable solubilizer and matrix-former in different studies [60,61], but it can be observed that its use in high amounts and alone, was not an appropriate choice, as higher temperatures were needed for the mixture to melt, thus causing the AMH degradation. Different grades of PEG were also chosen as polymer-carriers, due to their hygroscopic semi-crystalline character and excellent plasticizer properties [62]. Kolliphor^®^ P188 has the advantages of a low melting point (below 60 °C), great thermal binder ability, and good plasticizing and solubilizing effects [14,63,64]. Considering the hot-melt extrusion process evolution and the appearance of the obtained extrudates and powders, the qualitative and quantitative selection of the polymers seems to be properly conducted.

The appearance of the SDs is not influenced by the type of polymer used, rather their morphology is dependent on the grinding process.

SD4 manifested an excellent flowability and compressibility behavior that can be explained only by the correct proportion between polymers in the matrix which offered a certain softness to the powders, as SD4 optical microscopic images showed lower particle sizes in comparison to the other formulations obtained under the same conditions. The advanced grounding could be achieved only due to the plastic nature of the extrudates [65]. Still, in contrast, SD2 which contains only PEG 8000 has only a fair flow. SD3 where PEG 8000 is replaced by PEG 4000, maintaining the ingredients’ amounts, also displays just a fair flow. Moreover, Poloxamer 188 included in other formulations, such as SD5 or SD11, leads to good flowability, demonstrating its important plasticizing effect in the solid dispersions systems [66]. The high differences in flow and compression performances of the formulations prove the relevant influence of the polymer type and also their concentration-dependent plasticizing properties.

The obtained particle size distribution for solid dispersions revealed a similar profile for most of the studied formulations, except SD9 and SD10. These results are strengthening the idea that the grounding process is not influenced by the type of the polymer, and in these cases, the particles’ fineness is not impacting the flow behavior [67].

Adding the tablets excipients to the solid dispersions, the physical properties of the solid dispersions were enhanced, leading to a superior flowability and compressibility of all mixtures, regardless of the polymer nature. The significant diminishment in the powders density is the result of the inter-particulate bonds that were formed [68].

The particle dimensions of the composed powders are found to be in the < 180 µm range, but still, they presented a great flowing behavior. This comes in contrast to the well-established idea that small particles provide low flowability [69,70], but their performance in the compression process is much better, revealed by the characteristics of the final tablets. Again, it was demonstrated that the type of polymer does not influence the particle size distribution, and, moreover, the lack of interactions between the solid dispersions and the compression excipients was proved. 

The uniformity in the tablets’ particle size between the different series proved the accuracy of the formulation, especially the selection of a special type of magnesium stearate as a lubricant, as the results demonstrate that the compression powders did not adhere to the punches or the dyes.

Additionally, the mass uniformity of all formulations indicates the materials’ optimal flowability, confirming the results achieved in the preformulation studies. The small differences in sizes and weight registered between the tablets’ batches are obviously due to the influence of the polymer type.

Furthermore, the same type of polymer influence is noticeable also in the hardness values, as it is the only element modified in the tablets’ formulation and manufacturing process. The highest hardness is presented by F2 which contains only one polymer, PEG 8000. Surprisingly, when combining PEG 8000 with Soluplus^®^ in a 3:1 mass ratio (F10), a significant decrease in hardness is observed. 

Furthermore, in the case of mixing PEG 1500 with Soluplus^®^ maintaining the same 3:1 mass ratio (F8), a drastic diminishment in the hardness, to 78 N, was registered. 

The influence of the polymer type is best detected in the disintegration test, where the obtained values are quite different between the studied formulations. The use of PEG 1500 alone or combined in various proportions with Soluplus^®^ (F6–F8) leads to rapid disintegration. In contrast, the mixture of PEG 4000 and Poloxamer 188 in equal proportions (F3) determined the increase in disintegration time to 120 s. The use of Poloxamer 188 alone and a 3:1 combination of PEG 4000 and Soluplus^®^ (F5 and F9) also generates a prolonged disintegration time for the tablets.

F4 presents the highest dissolution rate after 60 min, even if it did not show the lowest disintegration time or hardness. In the preformulation studies, F4 displayed excellent flowability and compressibility, leading to a 4.15 mm thickness, a value higher than the other formulations. Correlating the results, the meaningful influence of the pre-compression parameters of the complex powders on the final performance of the product is noticed.

F3 and F5 also exhibited a dissolution rate of over 95%, after 60 min, proving there is no accordance between disintegration time (120 s for F3 and 160 s for F5) and the amount of drug released. 

However, F9, which also disintegrates in 160 s, released 91.7% of AMH after 60 min and only 93% after 90 min, presenting the lowest performance of all batches, and it even displayed a reduced hardness.

Subsequently, after 90 min, F8 exposed the highest dissolution rate of 99.8%, meaning that the 3:1 weight ratio of PEG 1500 and Soluplus^®^ matrix assures the complete release of AMH in a reasonable time for immediate-release tablets. After 90 min, F10 (with a 3:1 mass ratio of PEG 8000 and Soluplus^®^) also released a high AMH proportion of 98.4%. As expected, F4, too, exhibited a good dissolution rate of 97.96%, at 90 min. 

## 5. Conclusions

The obtained results on the tablets’ quality proved the accuracy of the formulation, consisting of the polymer-carrier types and selection of adequate proportions for solid dispersions, and by choosing the suitable sorts and amounts of excipients for the tablets. The direct compression process was well-conducted, and the parameters were correctly established, as all series of tablets present corresponding pharmacotechnical and in vitro biopharmaceutical attributes. The physico-chemical characterization using FTIR analysis demonstrated the existence of possible interactions between the drug and the used excipients. The XRD analysis revealed a reduction in drug crystallinity within the SDs. Even if all formulations lead to satisfactory pharmaceutical products, the influence of the polymer types and the amounts was demonstrated by all performed studies. Considering the registered release profiles, the most important test that can predict tablets’ in vivo behavior, the increase in dissolution rate, when using Poloxamer 188 in solid dispersions formulations, was demonstrated. Similarity factor matrix between formulations showed, on the other hand, that when comparing the data up to 45 min in dissolution, the best improvement in dissolution rate and profile is the formulation F9 that uses a mixture of Soluplus® and PEG4000 at a ratio of 25/75. Additionally, the RSD of the formulation F9 was better than the commercial formulation.

## Figures and Tables

**Table 1:** The solid dispersions formulations and the processing temperature.

Ingredients	Quantity (mg)											
	SD1	SD2	SD3	SD4	SD5	SD6	SD7	SD8	SD9	SD10	SD11	SD12
AMH	50	50	50	50	50	50	50	50	50	50	50	50
PEG 4000	100	-	50	-	-	-	-	-	75	-	-	-
PEG 8000	-	100	-	50	-	-	-	-	-	75	-	-
Poloxamer 188 (Kolliphor^®^ P188)	-	-	50	50	100	-	-	-	-	-	75	-
PEG 1500	-	-	-	-	-	100	50	75	-	-	-	-
Soluplus^®^	-	-	-	-	-	-	50	25	25	25	25	100
Total	150	150	150	150	150	150	150	150	150	150	150	150
Processing temperature (°C)	90	90	90	90	90	90	100	120	110	130	110	140

**Table 2:** Powders flowability scale.

CI (%)	Free Flow	HR
5–10	Excellent (free flow of granules)	1.00–1.11
10–15	Good (free flow of powdery granules)	1.12–1.18
16–20	Fair (medium flow of powdery granules)	1.19–1.25
21–25	Passable (very fluid powders)	1.26–1.34
26–31	Poor (fluid cohesive powders)	1.35–1.45
32–37	Very poor (cohesive powders)	1.46–1.59
>38	Very, very poor (very cohesive powders)	>1.60

**Table 3:** Oral immediate-release tablets containing AMH SD.

Ingredients	Quantity (mg)										
	F1	F2	F3	F4	F5	F6	F7	F8	F9	F10	F11
AMH	50	50	50	50	50	50	50	50	50	50	50
PEG 4000	100	-	50	-	-	-	-	-	75	-	-
PEG 8000	-	100	-	50	-	-	-	-	-	75	-
Poloxamer 188 (Kolliphor^®^ P188)	-	-	50	50	100	-	-	-	-	-	75
PEG 1500	-	-	-	-	-	100	50	75	-	-	-
Soluplus^®^	-	-	-	-	-	-	50	25	25	25	25
Microcrystalline cellulose (VIVAPUR^®^ 302)	340	340	340	340	340	340	340	340	340	340	340
Sodium starch glycolate (EXPLOTAB^®^)	5	5	5	5	5	5	5	5	5	5	5
Magnesium stearate (LIGAMED^®^ MF-2-V)	5	5	5	5	5	5	5	5	5	5	5
TOTAL	500	500	500	500	500	500	500	500	500	500	500

**Table 4:** Volumetric characteristics for the hot-melt extrusion SD.

Parameter	Formulation Code										
	SD1	SD2	SD3	SD4	SD5	SD6	SD7	SD8	SD9	SD 10	SD11
V0	29 ± 0.12	29 ± 0.23	33 ± 0.19	30 ± 0.04	32 ± 0.15	18 ± 0.07	30 ± 0.33	31 ± 0.35	29 ± 0.28	30 ± 0.07	31 ± 0.13
V10	27 ± 0.17	26 ± 0.12	31 ± 0.22	29 ± 0.10	30 ± 0.21	16.5 ± 0.13	28 ± 0.26	28 ± 0.27	26 ± 0.23	27 ± 0.08	30 ± 0.20
V500	26 ± 0.07	24 ± 0.31	28 ± 0.17	27 ± 0.11	28 ± 0.27	15.5 ± 0.06	26 ± 0.19	27 ± 0.19	25 ± 0.14	25 ± 0.11	27 ± 0.14
V1250	25 ± 0.11	24± 0.28	27 ± 0.24	27 ± 0.09	28 ± 0.09	15.5 ± 0.18	26 ± 0.28	26 ± 0.34	25 ± 0.26	25 ± 0.16	27 ± 0.07
Bulk density (g/mL)	0.69	0.69	0.61	0.67	0.63	0.56	0.67	0.65	0.69	0.67	0.65
Tapped density (g/mL)	0.80	0.83	0.74	0.74	0.71	0.65	0.77	0.77	0.80	0.80	0.74
Carr Index (CI) (%)	13.75	16.87	17.57	9.46	11.27	13.85	12.99	15.58	13.75	16.25	12.16
Hausner’s ratio (HR)	1.16	1.20	1.21	1.10	1.13	1.16	1.15	1.18	1.16	1.19	1.14
Flowability	Good	Fair	Fair	Excellent	Good	Good	Good	Fair	Good	Fair	Good

**Table 5:** Volumetric characteristics for the direct compression blends.

Parameter	Formulation Code										
	F1	F2	F3	F4	F5	F6	F7	F8	F9	F 10	F11
V_0_	51 ± 0.08	54 ± 0.62	56 ± 0.34	50 ± 0.26	54 ± 0.45	55 ± 0.47	52 ± 0.22	55 ± 0.52	51 ± 0.33	53 ± 0.27	50 ± 0.57
V_1250_	44 ± 0.18	45 ± 0.41	46 ± 0.51	46 ± 0.39	46 ± 0.37	48 ± 0.62	46 ± 0.31	48 ± 0.27	45 ± 0.34	47 ± 0.13	45 ± 0.23
Bulk density (g/mL)	0.39	0.37	0.36	0.40	0.37	0.36	0.38	0.36	0.39	0.37	0.40
Tapped density (g/mL)	0.45	0.44	0.43	0.43	0.43	0.41	0.43	0.41	0.44	0.42	0.44
Carr Index (CI) (%)	13.33	15.99	16.27	6.97	13.95	12.19	11.62	12.19	11.36	11.90	9.09
Hausner’s ratio (HR)	1.15	1.18	1.16	1.07	1.16	1.13	1.13	1.13	1.12	1.13	1.10
Flowability	Good	Good	Good	Excellent	Good	Good	Good	Good	Good	Good	Excellent

**Table 6:** AMH SD oral immediate-release tablets characteristics.

Parameter	Formulation code										
	F1	F2	F3	F4	F5	F6	F7	F8	F9	F 10	F11
Diameter (mm)	12.1 ± 0.03	12.1 ± 0.25	12.0 ± 0.88	12.0 ± 0.10	12.0 ± 0.73	12.1 ± 0.34	12.0 ± 0.61	11.9 ± 0.48	12.0 ± 0.89	11.9 ± 0.62	12.1 ± 0.88
Thickness (mm)	4.13 ± 0.26	4.15 ± 0.08	4.08 ± 0.73	4.15 ± 0.17	4.09 ± 0.39	4.11 ± 0.55	4.13 ± 0.11	4.02 ± 0.48	4.15 ± 0.29	4.07 ± 0.61	4.16 ± 0.64
Weight (mg)	498.2 ± 0.65	504.0 ± 0.58	494.6 ± 0.67	504.0 ± 0.32	497.6 ± 0.62	502.8 ± 0.68	496.8 ± 0.44	492.1 ± 0.52	501.5 ± 0.63	493.4 ± 0.20	506.3 ± 0.72
Hardness (N)	82 ± 3.77	96 ± 4.16	79 ± 4.25	84 ± 1.38	81 ± 3.14	94 ± 4.63	89 ± 2.65	87 ± 5.07	78 ± 1.97	82 ± 4.33	95 ± 4.12
Friability (%)	0.10	0.10	0.10	0.10	0.20	0.10	0.20	0.10	0.10	0.10	0.10
In vitro disintegration time (seconds)	30 ± 3.18	40 ± 1.44	120 ± 5.27	40 ± 2.13	160 ± 5.78	10 ± 2.15	25 ± 1.88	10 ± 3.95	160 ± 2.69	30 ± 1.77	70 ± 4.21

**Table 7:** AMH SD oral immediate-release tablets characteristics similarity *f*2 factor matrix.

Similarity Factor *f*2 Between Formulations												
Formulation	F1	F2	F3	F4	F5	F6	F7	F8	F9	F10	F11	Commercial
F1		64.20	35.81	51.39	38.70	86.10	44.97	44.49	48.23	82.29	57.37	68.21
F2	64.20		41.30	60.11	44.88	58.99	37.98	37.26	40.12	64.77	46.42	73.71
F3	35.81	41.30		41.76	46.18	34.24	26.53	25.24	27.04	35.97	29.17	37.64
F4	51.39	60.11	41.76		40.63	48.53	35.86	33.76	37.46	55.10	40.42	53.05
F5	38.70	44.88	46.18	40.63		37.49	27.16	27.21	28.07	38.02	32.38	42.92
F6	86.10	58.99	34.24	48.53	37.49		46.84	46.95	50.45	74.47	61.72	63.78
F7	44.97	37.98	26.53	35.86	27.16	46.84		61.29	71.09	44.67	50.19	38.92
F8	44.49	37.26	25.24	33.76	27.21	46.95	61.29		65.64	43.29	56.52	39.44
F9	48.23	40.12	27.04	37.46	28.07	50.45	71.09	65.64		48.00	57.45	41.81
F10	82.29	64.77	35.97	55.10	38.02	74.47	44.67	43.29	48.00		55.04	65.99
F11	57.37	46.42	29.17	40.42	32.38	61.72	50.19	56.52	57.45	55.04		51.09
Commercial	68.21	73.71	37.64	53.05	42.92	63.78	38.92	39.44	41.81	65.99	51.09	

## Data Availability

Not applicable.

## Sample Availability

Samples of the compounds are available from the corresponding authors.
